# Paediatric Pilonidal Sinus Disease: Early Recurrences Irrespective of the Treatment Approaches in a Retrospective Multi-centric Analysis

**DOI:** 10.1007/s00268-023-07045-x

**Published:** 2023-05-18

**Authors:** Sophie K. M. Maasewerd, Maria-Christina Stefanescu, Tatjana T. König, Marie N. Engels, Stephan Rohleder, Martin Schwind, Andreas C. Heydweiller, Christina Oetzmann von Sochaczewski

**Affiliations:** 1 Klinik für Kinderchirurgie, St. Marien Hospital Bonn, Bonn, Germany; 2 Klinik und Poliklinik für Kinderchirurgie, Universitätsmedizin Mainz, Mainz, Germany; 3 Sektion Kinderchirurgie der Klinik und Poliklinik für Allgemein-, Viszeral-, Thorax- und Gefäßchirurgie, Universitätsklinikum Bonn, Bonn, Germany

## Abstract

**Background:**

Incidences of pilonidal sinus disease are rising. Guidelines rarely consider children and adolescents and evidence for their treatment is rare. The literature is divided on the choice of the preferable surgical procedure. Therefore, we aimed to assess recurrences and complications following different treatment approaches in our multi-centric cohort.

**Methods:**

We retrospectively assessed all patients treated for pilonidal sinus disease in the paediatric surgical departments of Bonn and Mainz between 01/01/2009 and 31/12/2020. Recurrences were defined according to the German national guidelines. The pre-specified analysis via logistic regression included the operative approach, age, sex, use of methylene blue, and obesity as independent predictors.

**Results:**

We included 213 patients, of which 13.6% experienced complications and 16% a recurrence. Median time to recurrence was 5.8 months (95% confidence interval: 4.2–10.3), which was slightly higher in children than adolescents (10.3 months, 95% confidence interval: 5.3–16.2 *vs.* 5.5 months, 95% confidence interval: 3.7–9.7). None of the investigated procedures, excision and primary closure, excision and open wound treatment, pit picking, and flap procedures had a decisive advantage in terms of complications or recurrence. Of the independent predictors, only obesity was associated to complications (adjusted odds ratio: 2.86, 95% confidence interval: 1.05–7.79, *P* = 0.04).

**Conclusions:**

We did not find a difference between the investigated procedures, but our analysis is limited by the small sample size in some subgroups. Our data corroborates that recurrences in paediatric pilonidal sinus disease occur early. Factors linked to these differences remain unknown.

## Introduction

Pilonidal sinus disease is on the rise, both locally [1–3] and globally [4, 5]. Its incidence rates are now exceeding those of inguinal hernia in the most affected age groups [6]. This development is paralleled by a decreasing age at diagnosis [1, 6]. National guidelines were developed in Italy [7], the USA [8], and Germany [9], but seldom mentioned children and adolescents specifically. The literature is divided on the procedure of choice for paediatric pilonidal sinus disease: Some favour resection and primary midline closure [10–13], others excision and open wound treatment [14, 15], endoscopic [16, 17], minimally invasive [18–20] or flap procedures [21, 22]. Despite these numerous reports on a variety of treatment approaches, none of them was determined to be advantageous in a systematic review of surgical approaches to paediatric pilonidal sinus disease [23]. We therefore analysed all paediatric surgical patients treated in our departments with respect to complications and recurrences following surgery for pilonidal sinus disease with different surgical approaches using pre-specified predictors in pre-specified analyses.

## Material and methods

### Patient population

We retrospectively identified all surgically treated patients in the paediatric surgical departments of the German cities Bonn and Mainz between the 1 January 2009 and the 31 December 2020. Inclusion criteria for our study were patient age below 18 years and diagnosis of pilonidal sinus disease with (ICD-10-GM L05.0) or without (ICD-10-GM L05.9) an abscess. Exclusion criteria were either no surgical treatment or only incision and drainage of an abscess if no data beyond that procedure was available, age above 18 years despite being treated in a paediatric surgical department, and diagnosis of a dermal sinus. Postoperatively, patients were routinely followed until their wounds were healed enough in order to be subsequently treated by the primary care physician.

### Data collection

A pre-specified, standardised abstraction form was developed and used by trained chart abstractors that collected the clinical information according to a specific procedural manual. Occasionally, the results were cross-checked by other investigators in order to ensure the quality of the retrospective chart abstraction. Before distribution to all departments, a pilot study was conducted at the *Universitätsklinikum Bonn* in order to test the suitability of the in- and exclusion criteria and the operationalisation of the included variables. We collected information on age, sex, presence of a concurring abscess, height and bodyweight, pre-existing medical conditions, previous operations for pilonidal sinus disease, type of surgical procedure, intraoperative use of methylene blue or one of its alternative dyes, use of negative pressure wound therapy, length of hospital stay, occurrence of a postoperative complication, and recurrence including the time until the patient presented again. Reporting of the study adhered to the STROBE-guidelines [24].

### Definitions

Obesity was defined according to body mass index above the 97 percentile of the German reference cohort [25, 26]. The onset of puberty was defined as an age of 12.8 years according to present German reference data [27] and the whole patient cohort was separated into pre-pubertal children and adolescents according to this age-limit to account for the relevance of puberty [28]. The remaining quantitative variables were treated as such. Complications included wound infections, impaired wound healing [29], and wound dehiscence in all procedures including any form of wound closure. Recurrence was defined as a postoperative presentation with at least one new porus accompanied by symptoms, based on the definition in the German guidelines [9]. Pre-specified predictors of a negative outcome were chosen based on reports from the literature: Age [30], sex [30, 31], use of methylene blue [32], and obesity [31, 33, 34] were previously suggested to influence recurrence in paediatric patients. They were thus included in the regression analyses besides the different surgical procedures. Patients on whom information to calculate the body mass index were not available, were excluded from the regression analysis, as obesity was among the pre-specified predictors.

### Statistical analysis

All statistical analyses were conducted in R (version 3.5.3) [35]. Pre-specified predictors of complications and recurrences were assessed via logistic regression. In pre-pubertal children, maximum penalised likelihood was used via the brglm-package (version 0.7.2) [36, 37], because the single diverse patient in this cohort caused quasi-complete separation resulting in non-convergence of the maximum likelihood algorithm. Confidence intervals for medians were calculated via the MedianCI-function from the DescTools-package (version 0.99.35) [38]. During peer-review, exploratory analyses of the additional predictor abscess in the regression analysis, length of hospital stay and type of anaesthesia were included: The former consisted of pairwise-comparisons by Mood’s test and the latter of pairwise-comparisons by Fisher’s test. The false discovery rate of these exploratory analyses was controlled by the Benjamini–Hochberg procedure [39].

## Results

We identified 281 eligible patients during the twelve-year study period. Two of them were 18 and 20 years old, another two had a spina bifida occulta and were misclassified. The legal guardians of three patients refused a surgical treatment and nine patients were treated by incision and drainage of their abscesses without additional follow-up at the treating department. Another 52 patients, the vast majority of them were pre-pubertal children, had asymptomatic disease at the time of presentation and opted for watch and wait. We thus included 213 patients in our study, of which 77 were pre-pubertal children and 136 were adolescents (Table 1). Information on obesity was available for 190 patients, who were included in the regression analysis.

**Table 1:** Relevant properties of the patient cohorts. The 95% confidence interval of the median was calculated using the *t*-distribution

	Whole cohort	Children	Adolescents
	[*n* = 213]	[*n* = 77]	[*n* = 136]
Age (median, IQR) [years]	14.1 (3.7–15.6)	1.5 (0.7–4.2)	15.2 (14.2–16)
Females: Males: Diverse (*n*)	102: 110: 1	31: 45: 1	71: 65
Abscesses (*n*, %)	101 (47.4)	8 (10.4)	93 (68.4)
Obesity (*n*, %)	45 (23.6) [*n* = 190]	15 (22.4) [*n* = 67]	30 (24.4) [*n* = 123]
Use of methylene blue (n, %)	76 (35.7)	9 (11.7)	67 (49.3)
Primary closure (*n*, %)	83 (39)	70 (90.9)	13 (9.6)
Open wound treatment (*n*, %)	97 (45.5)	5 (6.5)	92 (67.7)
Pit picking (*n*, %)	8 (3.8)	2 (2.6)	6 (4.4)
Karydakis flap (*n*, %)	18 (8.5)	0	18 (13.3)
Bascom cleft lift (*n*, %)	3 (1.4)	0	3 (2.2)
Limberg flap (n, %)	2 (0.9)	0	2 (1.5)
Dufourmentel flap (n, %)	2 (0.9)	0	2 (1.5)
Complications (n, %)	29 (13.6)	5 (6.5)	24 (17.7)
Recurrences (*n*, %)	34 (16)	3 (3.9)	31 (22.8)
Time to recurrence (median, 95% CI) [years]	0.48 (0.35–0.86)	0.86 (0.44–1.35)	0.46 (0.31–0.81)

*IQR* = interquartile range, *CI* = Confidence interval

Our data supported the *a priori* decision to separate the whole cohort into two groups: Pre-pubertal children and adolescents. The age distribution within the different groups was quite homogenous (Table 1). Only 12 adolescents had been operated for a pilonidal sinus before and 49 patients had any comorbidity. Median lengths of hospital stay were 2 days (95% confidence interval: 2–2 days) in children and 4 days (95% confidence interval: 4–5 days) in adolescents. In an exploratory analysis, median length of hospital stay was shorter for pit picking compared to primary closure and open wound treatment (Table 2). Patients that had pit picking were more often managed as outpatients and frequently had local or regional anaesthesia (Table 2). The use of negative pressure wound therapy was not associated to an increased length of hospital stay.

**Table 2:** Properties of the cohort separated by surgical approach. The 95% confidence interval of the median was calculated using the t-distribution. Confidence intervals for medians were not calculated for groups with less than five events

	Primary closure	Open wound treatment	Pit picking	Karydakis flap	Bascom cleft lift	Limberg flap	Dufourmentel flap
Length of hospital stay (median) [days]^γ^	2 (95% CI: 2 – 2)	5 (95% CI: 4 – 5)	0.5 (95% CI: 0 – 2)^ε^	3 (95% CI: 2 – 4)	4	14.5	8.5
Outpatients [*n*, %]	6 (7%)	3 (3%)	4 (50%)	0	0	0	0
Anaesthesia^δ^							
General [*n*]	83^ζ^	97^η^	4^θ^	15	3	2	2
Spinal [*n*]	0	0	1	3	0	0	0
Local [*n*]	0	0	3	0	0	0	0
Abscess [*n*, %]	10 (12%)	77 (79%)	4 (50%)	6 (33%)	2	2	0
Complication [*n*, %]	5 (6%)	12 (12%)	1 (13%)	6 (33%)	2	1	2
Recurrence [*n*, %]	2 (2%)	23 (24%)	4 (50%)	3 (17%)	1	0	1
Time to recurrence (median) [months]	3.7	6.4 (95% CI:4.2 – 16.8)	5.8	6.5	4.4	–	3.6

*CI* = confidence interval; *γ* Corrected statistical significance level q*_1_ = 0.017; *δ* Corrected statistical significance limit q*_2_ = 0.042; *ε* *P* = 0.012 vs. primary closure and *P* < 0.001 vs. open wound treatment; *ζ* *P* < 0.001 vs. pit picking and *P* = 0.005 vs. Karydakis flap; *η* *P* < 0.001 vs. pit picking and *P* = 0.003 vs. Karydakis flap; *θ* *P* = 0.034 vs. Karydakis flap

Excision and primary midline closure was the dominant procedure in children, while it was excision and open wound treatment in adolescents (Table 1). Complications occurred in 13.6% of the whole cohort and were more prevalent in adolescents with 17.7% (Table 1). This was similar for recurrences, which occurred in 3.9% of children, but in 22.8% of adolescents (Table 1). Time to recurrence was short with a median time of 0.46 years with an upper limit of only 0.81 years of the 95% confidence interval in adolescents. Median time to recurrence in children was 0.86 years with an upper limit of the 95% confidence interval of 1.35 years (Table 1). The longest time to recurrence in our cohort was 4.96 years. Time to recurrence did not differ between the procedures (Table 2).

**Fig. 1:**
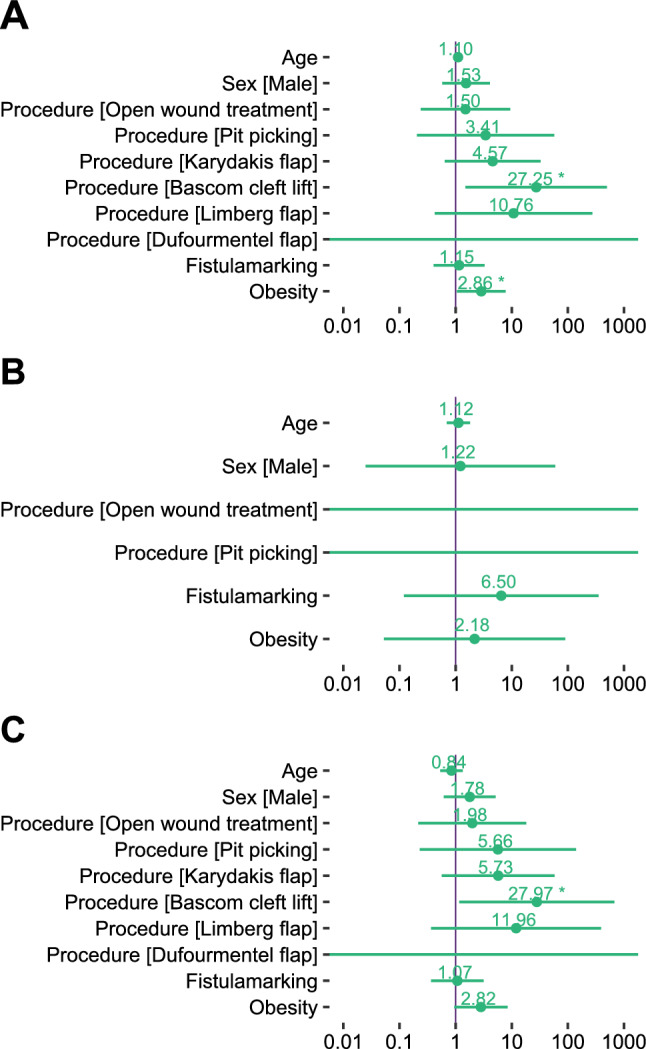
Pre-specified logistic regression analysis of complications. Presented are adjusted odds ratios with a logarithmic scale. Primary midline closure is the reference category for the regression of surgical procedures. **a** Logistic regression of complications in the whole cohort (*n* = 190). Bascom’s cleft lift is statistically significant with *P* = 0.026 and obesity with *P* = 0.04. **b** Logistic regression of complications in children (*n* = 67). **c** Logistic regression of complications in adolescents (*n* = 123). Bascom’s cleft lift is statistically significant with *P* = 0.04

Obesity was a relevant predictor for complications with an increased adjusted odds ratio of 2.86 (95% confidence interval 1.05–7.79, *P* = 0.04) in the whole cohort of 190 patients (Fig. 1a). In the subgroups of children (Fig. 1b) and adolescents (Fig. 1c) this association was not present. Other pre-specified predictors’ associations to complications were less compatible with the data, but this analysis is limited by the small number of procedures in many groups and the resulting wide confidence intervals (Fig. 1).

**Fig. 2:**
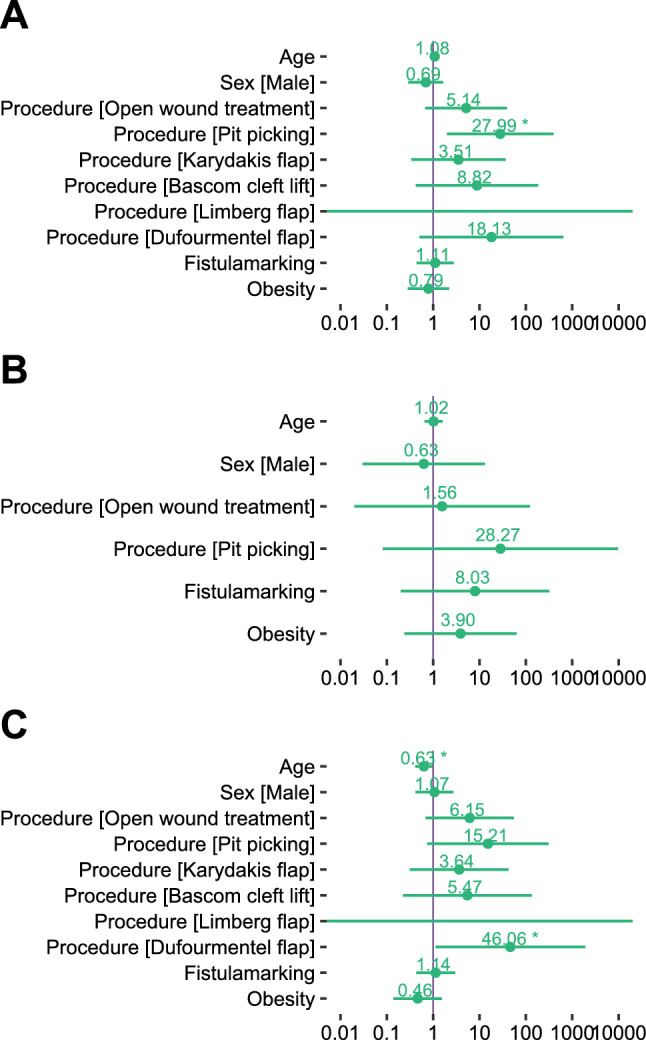
Pre-specified logistic regression analysis of recurrences. Presented are adjusted odds ratios with a logarithmic scale. Primary midline closure is the reference category for the regression of surgical procedures. **a** Logistic regression of recurrences in the whole cohort (*n* = 190). Pit picking is statistically significant with *P* = 0.014. **b** Logistic regression of recurrences in children (*n* = 67). **c** Logistic regression of recurrences in adolescents (*n* = 123). Age is statistically significant with *P* = 0.035 and the Dufourmentel flap with *P* = 0.044

This was similar for recurrences: In the whole cohort, pit picking seems to be disadvantageous with an increased odds ratio of 27.99 (95% confidence interval: 1.98–395.28, *P* = 0.014) (Fig. 2a). However, this was not the case in the subgroups of children (Fig. 2b) and adolescents (Fig. 2c). Due to the low number of procedures in many groups, the resulting confidence intervals for the other independent predictors are quite wide and thus no definitive answers could be provided (Fig. 2).

Exploratory analyses including abscesses as an additional covariate did not change the regression coefficients, but decreased the relative quality of the statistical models based on an increased Akaike information criterion.

## Discussion

Increasing incidences of pilonidal sinus disease [4] are not limited to adults, but can also be observed in children and adolescents [1]. In adults, primary midline closure results in unacceptably high recurrence rates compared to excision and open wound treatment [40, 41], whereas it is still considered the treatment of choice for children and adolescents in several reports [10–13]. Among the treatment alternatives, reports advocating their use can be found for almost every method: The conventional excision and open wound treatment [14], endoscopic [16], minimally invasive [18], and flap procedures [21]. Due to the contrasting evidence, we aimed to assess whether any surgical treatment would be favourable in children or adolescents.

However, we were not able to demonstrate the association of a certain procedure to an increased or decreased chance for recurrence or complications. The exceedingly large confidence intervals of the independent predictors in our analyses indicate that our sample size might still be too small to gather reliable results. This is emphasised by the fact that we found statistically significant results only in subgroups with a small number of patients. These results are likely to have been influenced by the small number of events. For example, the statistically different chance of recurrence between pit picking and primary closure can be attributed to the small number of recurrences in children.

Our subgroups were based on a pilot study that was conducted in order to test the feasibility of our planned investigation in a subset of patients, as recommended elsewhere [42]. Combing different approaches, e.g. the flap procedures, into a single category would likely not provide additional information, as the number of patients would still be small. However, it would alter the statistical accuracy of the test and thus affect the confidence in the results [43]. In this case, our study would be accompanied by a higher risk of outcome bias in evidence synthesis using the criteria employed by a recent systematic review [23]. Despite the small patient numbers in several subgroups, which may impede generalisability of our results due to the large numbers of patients treated by excision and open wound healing, our study might still add some information to the literature: Due to its multi-centric approach and the considerably large number of patients treated by excision and open wound treatment, our study might provide a low bias reference for sample size calculations.

Another important aspect of our study is the very short time to recurrence: A cut-off of 2% yearly recurrence rate of pilonidal sinus disease following surgery has been suggested in adults [40]. In our cohort, recurrences occurred quite early compared to what would be expected in adults. A similar pattern of recurrences had been observed in registry data [30] and single-centre studies from different regions: [10, 13, 14, 16, 19–21, 34] Median time to recurrence ranged from 2.9 months [16] to 12 months [20]. Surprisingly, this was neither addressed in original studies [10, 13, 14, 16, 19–21, 34] nor in systematic reviews [23, 44]. Recently, this issue has been raised, because the causative factors remain unknown [18]. Studies whose cohorts included patients with recurrences or report their exclusion [18, 19, 21, 45] indicate a significant burden of disease, as up to 40% [45] of the cohort had recurrences. Therefore, it has been suggested to stick to repeated minimally invasive procedures for pilonidal sinus disease in children [46]. Consequently, a recent treatment-algorithm suggested to reserve flap procedures for multiple recurrences or extensive disease [47].

There is a portentous clinical consequence of early and repeated recurrences in paediatric pilonidal sinus disease: If the causative factors of this “paediatric pattern of recurrence” are unknown, we cannot determine the time to switch from the paediatric treatment approach to the adult one. Our large multi-centric cohort corroborated that recurrences in children and adolescents occur early. Future research should thus aim to identify factors responsible for this different “pattern of recurrence” in minors. It might be tempting to speculate that identifying these driving factors could also influence treatment success in young adults. It is unlikely that a patient’s 18^th^ birthday is the factor that would eliminate the “paediatric pattern of recurrence”.

In our institutions, we did not employ recommendations on hair removal, especially not laser hair removal, which was consequently not assessed in our study. Following the German guidelines [9], neither a positive nor a negative recommendation can be made. Thus, laser hair removal might only be considered for selected patients who experienced multiple recurrences [9]. Laser hair removal in adolescents has first been described by *Lukish* and co-workers [48] and gained momentum in the postoperative care of adolescents after two promising retrospective reports [21, 49]. Based on a prospective observational feasibility study [50], a randomised-controlled trial has been undertaken [51], whose estimated primary completion date was the end of last year, so we might expect its results soon. Recently reported treatment protocols from the USA include laser depilation either if hirsutism was present [45] or for every patient [52], whereas it was uncommon on the continent [18, 22, 53].

Relevant limitations of our study are the small sample size in some subgroups and its retrospective design that is inevitably associated with bias, although we tried to reduce it as much as possible by adhering to procedural precautions [42]. Guideline implementation might also have introduced additional bias, because prophylactic resections of asymptomatic disease were common before the first version of the German guideline discouraged this practice in 2014 [54]. Since then, watch and wait was the strategy of choice in our departments, although several parents still preferred prophylactic resections. Another issue is the definition of puberty based on reference data instead of individual onset of puberty, e.g. by the Tanner scale, which is likely to have resulted in some misclassifications. It is also likely that we missed some recurrences, because patients were treated in an adult surgical department after their eighteenth birthday.
